# Getting off the carousel: Exploring the wicked problem of curriculum reform

**DOI:** 10.1007/s40037-017-0371-z

**Published:** 2017-09-26

**Authors:** Lorraine Hawick, Jennifer Cleland, Simon Kitto

**Affiliations:** 1 Institute of Education for Medical and Dental Education, School of Medicine and Dentistry, University of Aberdeen, Aberdeen, UK; 2 Department of Innovation in Medical Education, Office of Continuing Professional Development, University of Ottawa, Ottawa, Canada

**Keywords:** Wicked Problem, Curriculum Reform, Case Study, Undergraduate Medical Education

## Abstract

**Introduction:**

Making substantial changes to the form and delivery of medical education is challenging. One reason for this may be misalignment between existing conceptualizations of curricula and curriculum reform in medical education, with the former perceived as ‘complex’ yet the latter as linear. Reframing curriculum reform as a process-driven, complex entity may enhance the possibility of change. To explore the utility of this approach, we carried out an exploratory case study of curriculum reform in a real-life context.

**Methods:**

We used a qualitative case study approach. Data were collected from 17 interviews with senior faculty involved in curriculum reform in one medical school plus document analysis of approximately 50 documents and files, to provide background, context, and aid triangulation.

**Results:**

Data coding and analysis was initially inductive, using thematic analysis. After themes were identified, we applied the ‘wicked problem’ framework to highlight aspects of the data. This paper focuses on two main analytic themes. First, that multiple players hold different views and values in relation to curriculum reform, resulting in various influences on the process and outcomes of reform. Second, ‘solutions’ generate consequences which go beyond the anticipated advantages of curriculum reform.

**Discussion:**

This is the first empirical study of curriculum reform in medical education which uses the wicked problem framework to conceptually illuminate the complex processes which occur in relation to reform. Those involved in reform must be reflective and attentive to the possibility that persistent and emerging challenges may be a result of wicked problems.

## What this paper adds

Making substantial changes to the form and delivery of medical education can be challenging. One reason for this may be that medical curriculum reform is often seen as linear but yet medical curricula themselves are complex and messy. By exploring the processes of curricular change in-depth, in one context, using the ‘wicked problem’ framework we were able to tease out multiple social and cultural factors influencing curriculum reform, as well as unpredictable and unintended consequences of reform. Acknowledging this complexity may aid those involved in change to manage uncertainty, as well as questioning and considering ‘issues’ productively rather than reproductively.

## Introduction

In the medical education literature there is a plethora of interest and research activity in curriculum reform [1–7]. However, this activity often results in repetition of sameness with little actual reform. Whitehead and colleagues [8] conducted an analysis of prominent recurrent themes in the North American medical education literature, focusing on the discourses of the good doctor from 1910–2010. A series of recurring themes were identified, including the need to avoid over-specialization, the importance of generalism, and the need to broaden criteria for medical student selection. Analysis of these recurring themes identified a prominent and recurrent discourse of ‘new.’ This discourse places focus on the future, ignores the ongoing historical nature of issues, suggests a sense of urgency and enables the proposal of modest solutions (P. 755). In analyzing the language used by medical educators to describe these recurring calls for change, Whitehead and colleagues [8] use the metaphor of a ‘carousel of ponies’ to illustrate that the returning themes of curriculum reform are like ponies circling around yet again on the curricular carousel, in the continual rediscovery of discursive ‘truths’ in medical education. This ‘carousel’ seems to be due to a combination of recurring themes underpinning the need for reform, a focus on the future with little acknowledgement of the historical nature of issues, and use of (relatively speaking) ‘quick fixes’ to address complex curriculum issues [9, 10].

How can medical education get off this carousel? We propose that key to doing so is to explicitly acknowledge an implicit disconnect. Medical curricula, indeed curricula more widely, are understood to be complex entities [9–12]. However, in contrast, empirical studies of curricula change in medical education are often located in a paradigm of simplification [13], where reform is conceptualized as linear, a response to practical needs, overcoming uncertainty and ambiguity, and formulating clear and accurate diagnoses (and hence simple solutions). Common approaches to curricular change (e. g [14, 15]). reflect this linearity. But questions have been raised as to whether these linear approaches to evaluating change are sufficient given the complex nature of curricula [10, 16, 17].

A fresh perspective may be useful to address this gap. To highlight how curriculum reform is a process rather than an outcome, rather than just focusing on ‘did it (the change(s)) work’ [2, 3, 6, 18, 19], it would be useful to also think about ‘how did it work’ and ‘what have we learned’ (from the process of reform). Those involved in curriculum reform will then change their perspective from one that is relatively simplistic and linear (and maintains the circling round and round), which in turn will help to slow down or get down from Whitehead et al.’s carousel [8].

To that end, our aim in this study was to produce a rich analytical description of the complex processes which occurred during a curriculum reform. In doing so, we hope to give a new perspective on reform and extend knowledge in the field.

## Methods

We conducted a qualitative study, using an exploratory case study design – an empirical inquiry that investigates a contemporary phenomenon within its real-life context [20] to scrutinize the process of curriculum reform at one UK medical school. Using a case study approach allowed us to answer ‘how’ and ‘why’ type questions, while taking into consideration how curriculum reform is influenced by the context within which it is situated [21].The study was underpinned by a social constructionist epistemology [22].

### Context

The setting for the study was a medium-sized (approximately 170 students per cohort), state-funded medical school in the UK. The aim of the reform was to move away from a ‘traditional’ style curriculum format where the first two years of the five-year medical program were theory and science based and the subsequent three years were more clinically focused, towards a more blended curriculum with emphasis on student-centred learning, fewer lectures, more small group teaching, increased use of clinical cases, and integration of clinical and basic science teaching and learning.

A number of key stakeholders were involved in the delivery of the medical program, and hence the reform. They included: anatomists, physiologists and biomedical scientists; family doctors (general practitioners) and hospital doctors from 26 clinical specialties; university staff and clinical staff seconded in to support the reform process on time-limited sessional contracts. As with all medical schools in the UK, the program under reform was, and still is, regulated by the General Medical Council, who set the learning outcomes required of medical students when they graduate and the teaching and assessment standards that medical schools must meet [23, 24].

### Data collection

The primary data collection method employed was semi-structured interviews. After obtaining ethical approval and appropriate institutional consents, purposive sampling strategy [25] was employed in order to identify relevant individuals who had specific knowledge and understanding of the reform plans and processes. We specifically targeted members of the Curriculum Reform Steering Group, who were central to planning and implementing the reform, with the view that these individuals would be able to provide credible information about the reform process [25]. They included senior medical school faculty, physicians, scientists and social scientists.

Recruitment was conducted via email. Positive responses were followed up by email providing more information about the study, and a convenient time and place for a face-to-face interview. Those who agreed to take part were invited to attend a one-to-one interview, the questions for which were developed by drawing on the literature on curriculum reform [8, 26–28]. We explored participants’ perspectives on the aims and objectives of the reform, and identified the factors which were influential in driving and planning change. Some key individuals also took part in a follow-up interview 4–6 months later, to explore themes generated and identified in the initial data further. Seventeen individual interviews were carried out with eleven senior Faculty (six male, five female). Six participants were interviewed twice to explore issues which had emerged in the first interview in more depth. The median interview length was 43 min (ranging from 26–55 min). All interviews were conducted by the same person (LH).

Document analysis was also undertaken to augment and support interview data analysis [29]. The aim of document analysis was to seek convergence and corroboration through the use of different data sources and methods [30, 31], to uncover meaning, develop deeper understanding and gain insights relevant to the study [32]. Document sources were from reports and minutes from curriculum reform steering group meetings and various sub-group meetings. These were internal groups (commissioned locally from within the medical school), assigned to make decisions on what to include, exclude, improve and change in specific parts of the curriculum.

The first author reviewed approximately 50 electronic and hard copy documents and files, positioned them in context and chronological order, then coded them for analysis. These included: meeting minutes, scribed action points from reform planning events, reform proposals, discussion papers and copies of presentations given to health service stakeholders.

### Data analysis

All interviews were audio-recorded with participant permission, transcribed for analysis, and entered into NVIVO 10 qualitative data management software. Data coding and analysis of transcribed interviews was inductive, using thematic analysis to generate an initial coding scheme [33]. Guided by interview data emergent themes, documents were subjected to a directed content analysis [34] in order to contextualize and elaborate on the interview data [31]. After the identification of themes and following further discussion, we moved beyond primary thematic analysis to a more theoretically directed approach to critically analyze how curriculum reform in this one context was being interpreted, shaped and affected [35]. Thus, the choice of theoretical frameworks evolved iteratively from the initial data analysis.

Rigour was ensured in a number of ways [36]. All interviews were undertaken by the first author to ensure continuity. We considered our positions and relationships with the data continually and critically [37]; in view of our different disciplinary backgrounds (LH is a registered nurse with many years working in the health service, now working within a medical school as a clinical educator; JC a clinical and occupational psychologist by background, working in medical education research; SK a medical sociologist with extensive qualitative research experience in medical education), research interests (a mutual interest in the changing shape and influences on undergraduate medical education and curriculum, but often working from different theoretical perspectives and different preferences in methodological approaches) and how these might have shaped our co-construction of the data. Preliminary data analysis was shared and discussed with social scientists and medical educators outside the research team to explore if the findings seemed credible and reasonable.

### Ethics permission

We obtained ethical approval for this study from the University Ethical Research Board, where this study was undertaken (not identified here to maintain anonymity of study site). The study was carried out in accordance with the Declaration of Helsinki, there was no potential harm to the participants, the anonymity of participants was guaranteed and the informed consent of participants was obtained.

### Theoretical framework

We used ‘wicked problems’ [38], as a conceptual lens to help us analytically describe the key aspects of reform process identified via interviews and documents. ‘Wicked’ problems are complex in nature, have innumerable causes associated with multiple social environments and actors with unpredictable behaviour and outcomes, making them difficult to define or even resolve. They are divergent and emergent problems [39] which are highly resistant to resolution [40], characterized as issues that are continually evolving, have no single solution that applies in all circumstances and where solutions can only be classified as better or worse, rather than right or wrong [38]. Wicked problems tend to be intractable and elusive because they are influenced by many dynamic social, cultural and political factors [38, 41]. The social complexity of wicked problems, their technical difficulties, and the interconnected nature of these problems with other problems, make them extremely difficult to manage [42]. Essentially, the wicked problem’s properties can be summarized as consisting of dynamic and interlocking issues that lack definitional clarity because multiple stakeholders in shifting social contexts have different interpretations and seek different outcomes [43].

Identification of wicked problems have progressed since Rittel and Webber (1973) first published on this concept [38]. In the fields of school teaching and higher education, defining educational issues as wicked problems has gained traction and in turn, advances a quest to understand ‘wickedity’ [38, 44–48]. Furthermore, as noted by Krause [48], universities (and by definition, schools of medicine) operate in a macro level policy environment requiring: accountability, funding arrangements, relationships with various governing bodies; quality assurance agencies, external and internal examiners, university leaders and so forth. At the micro level, they are also responsible for supporting divisional departments, as well as faculty members, ancillary staff and students. Thus, endeavours to achieve and demonstrate quality higher education while managing these various competing demands are indeed wicked [48].

Moreover, given that universities, medical schools, and curriculum processes and reforms are by their nature socially and culturally complex, it is highly likely that a large number of the current and forthcoming challenges will have wicked characteristics. Drawing upon the concept of ‘wicked problems’ [38], fresh insights can be generated into why reform in undergraduate medical education is so challenging.

## Results

We focus on two broad themes which were present in the data: (1) Multiple voices from multiple contexts in the reform process, and (2) Curriculum reform as a wicked problem: Ambiguity and collateral damage. These were selected because they were predominant in the data and represent the utility of the conceptual framework in making sense of the data [49]. We have interwoven results and theory together, to illustrate the utility of the conceptual framework in explaining the empirical data [50]. Quotations have been included in this paper to aid confirmability, to help the reader follow the logic of the story.

### Multiple voices from multiple contexts in the reform process

There were many different stakeholders and a diversity of views about the reform, ranging from reform being a *threat* (unnecessary and undesirable), an *opportunity*, or a *logistical challenge*. Thus, different parties had different but equally well-defined ideas about what the problem actually was, indicating different values in play. These ranged from not favouring reform (‘*Dr X was pretty much obstructive about everything*’ (R5)), wishing to leave things alone, to remain the same, to protect the status quo. Because to consider making a change to what they believed to be working well was just too much to take on or threatening: ‘*So there were those that believed, why throw that away, because that’s working reasonably well?*’ (R1) and ‘*[the coordinator for that particular part of the curriculum] worked very hard to establish the course … therefore he was reluctant to lose the position of [his] block within the final year’* (R9). While some faculty seemed to be against reform in order to protect their existing timetable allocation (‘*there was a bit of that territorial stuff going on* …’ (R7)), others perceived the reform to be a means of increasing their curriculum time, as an opportunity to include more teaching related to their particular teaching specialty within the medical program: ‘*The tendency for everyone was to want to have more for their subject*’ (R1).

It was also clear that the medical school as an institution did not exist in a vacuum, rather it worked in partnership with the local health service providers who provided the clinically based teaching for medical students, and who also had views about the reform. Curriculum reform documents confirmed this and provided useful background information to help understand the context in which the reform was being conceived, planned and implemented. One participant’s perspective illustrated the logistical challenge:

And there’s the other big stakeholder, the NHS … they had a view as well, so that influenced our discussions about … mundane things like group allocation [for student’s clinical teaching], but that’s quite important … because you can have a great, fantastic educational strategy but, if you don’t have facilities, then it’s really difficult’ (R6).

In addition, there were often different solutions to the ‘same’ problem [38], where the ‘solutions’ ranged from being *obstructive, doing nothing*, to pushing to *protect or extend one’s own fiefdom*, t*o accepting the vision*, depending on the stance of the stakeholder.

As a result of these differing perspectives on the problem and the solution, it is improbable, if not impossible, to technically solve the wicked problem of curriculum reform in a way which satisfies all those involved: the solution to one aspect of a problem (e. g., giving X more teaching time in the new curriculum) will lead to another problem for someone else (e. g., how to squeeze everything in when there are only so many timetabled hours available). Indeed the data indicated that not everyone was happy with the reform process: *‘… people felt very hurt … I think about all the effort they were putting in and not being recognized … so a bit of trampling on feelings went on …*’ (R4). Moreover, each solution will change the system, not always in the linear predictable ways implied by the preceding illustration (which we explore later).

### Curriculum reform as a wicked problem: Ambiguity and collateral damage

The endpoint of the reform was imprecise – to move from one type of curriculum to another – and lacked clarity or clear conceptualization. Compounding this were governmental factors, such as the context in which reform was occurring; and an expectation of a document from the regulator setting out new standards and outcomes expected in medical graduates. This created a broader sense of unease within the reform steering group as to what they were preparing medical students for: ‘*I’m not sure I know what doctors are going to be [here] for in 20 years’ time*’ (R9). In other words, the goals of reform were not clear, simple or even linear, and hence a simple solution of ‘if we do this, then we will achieve our goal’ was impossible.

While it was clear in the data that there was a vast amount of planning and many fundamental elements of the curriculum reform received specific attention, there were also casualties. Some of these were immediate, some only emerged over time. For example, people were unsure exactly how to bring the family medicine team onside, to include this in the reform process. It (early family medicine teaching) seemed to be a wicked problem embedded in a bigger wicked problem, because it was difficult to solve [38]:

The [xxxx] course was not terribly well integrated but that was always going to be a bit of a long game … you have to pick the battles that you’re going to win and that was a difficult political battle to win. (R6)

While this quote and others (*‘they were just not interested … [in reform]’* (R4)) illustrates the difficulty of pinpointing why something may not emerge as originally hoped, it is clear that this casualty was due to a number of intersecting leadership and values issues. For example, this course was not ‘terribly well integrated’ and had a weak power base from which to fight for more time/resources: ‘*they had their own political difficulties to deal with … their tutors had a very firm idea of what should be taught … and their course was not popular at that time …*’ (R6). Archived minutes recorded frequent discussions about the ‘topic’ of family medicine, which emphasized the importance of close liaison with the family medicine group (in order to embed suitable learning objectives associated with the reformed curriculum). Nevertheless, in comparison with other curriculum reform activities, there was a paucity of reported progress in the area of family medicine.

The effects of this casualty continue on today, with attempts to integrate family medicine teaching constantly ongoing, reflecting other aspects of wickedity: that a wicked problem has a ‘no stopping rule’ [38] and to have any chance of actually solving a problem requires a causal explanation of the discrepancy between actual state and desired state [38].

## Discussion

To the best of our knowledge, this is the first empirical study to explore the process of curriculum reform framed using the wicked problem concepts. Our data demonstrated explicitly the multiple social and cultural factors which influenced reform, specifically highlighting the existence and impact of multiple voices and the unintended consequences of reform. It was clear from our data that myriad, intersecting factors and relationships contributed to the process of reform, not always in predictable or desirable ways.

Acknowledging this ‘wickedity’ may aid those involved in change to manage uncertainty, as well as questioning ‘issues’ more productively rather than *reproductively*. For example, rather than viewing the unexpected as problematic, and hence to solve or circumvent, reflection on such aspects of process could be useful in the thinking and practice of curriculum reform. Using the wicked problem framework has the potential to enable stakeholders and leaders to develop a different kind of thinking and a language to talk about why a particular problem is so difficult and challenging, to create a path to select, examine and interrogate the issues with more clarity than might be the case currently [46].

Our aim was to produce a rich analytical description of the complex processes which occurred during a curriculum reform. In doing so, we give a new perspective on the challenges and complexities associated with reform and to shed light on these intractably ‘wicked problems’. While it is not the remit or scope of this paper to offer extensive suggestions for solutions to curriculum reform wicked problems, it is notable that a different kind of leadership is required when trying to handle wicked problems; leaders are required who are not afraid to admit that they do not know everything [41]. Wicked problems require the transfer of authority from an individual leader to a collective of stakeholders because only collective and collaborative engagement can hope to address the problem [41].

One medical school may look very much like another superficially, leading to the temptation to lift a ‘solution’ from one context and roll it out in another. However, we know that particular curricula look different in different contexts [51, 52]. Acknowledging that each medical school has a unique space and place [16], and the relationships, dynamics and interactions between people and systems will vary because of context is crucial. In wicked problem terms this may be summed up by remembering that ‘solutions might be applied to seemingly familiar problems which are quite incompatible with them’ [38, P. 165].

The current study joins an existing conversation on this topic and indeed is tightly linked to the Whitehead et al. [8] ‘Captive on a Carousel’ paper. Many of the challenges experienced by the participants in our study resonated with Whitehead and colleagues work, where there had been historical calls for change and yet a sense of repetition in what required to be changed. Our findings add to this aspect of the conversation on curriculum reform, extending beyond the carousel metaphor by providing empirical data. Interestingly there are data from other education sectors (school and higher education generally) which indicate that curriculum reform does possess wicked characteristics [47].

The case study approach [29] is a good epistemological fit with the wicked problem framework as it allowed us to explore richness, complexity and context. However, we acknowledge that we looked at curriculum reform from one perspective only: that of the clinical and academic staff who were central to the reform processes. The views of other stakeholders, for example, clinical staff who supported students on placements may have enriched the data and/or foregrounded different themes. Our aim with a case study was to generate conceptual generalisability (not empirical generalizability) [53] which can now be assessed by others for its transferability and potential for applicability to other situations [54], such as to inform the design and conduct of future curriculum reforms and accompanying research. Note, to protect the anonymity of the locality and individual participants, we have given less detail about our context than might be considered standard in case study research.

The retrospective interview is an accepted method of knowledge construction which can contribute to the understanding of processes of changes in educational practice [55, 56] but can be criticized on the grounds of respondents’ memories fading with time. To address this, we used precise prompting to aid recall [57] and triangulated the interview data with document data analysis [58–60]. Because ‘no research is free of the biases, assumptions, and personality of the researcher and we cannot separate self from those activities in which we are intimately involved’ [61], we constantly considered our own positions in relation to the study and the data.

We chose to only present two broad categories of data here: broadly speaking those of multiple voices speaking from multiple contexts who hold different views and values in relation to the reform problem and process, and the unintended consequences of curriculum reform as a wicked problem. We did not report on other aspects of wickedity, such as the ongoing nature of reform present in our data because of word limits.

To conclude, curriculum reform demonstrates true complexity. This study highlights that one possible way of avoiding the trap of curriculum reform being part of a socio-cultural reproduction exercise is to focus on the process not just the outcome of change. Acknowledging this in terms of ‘wickedity’ may aid those involved in change to manage uncertainty, and reflect on challenges.
